# Characterization of the complete chloroplast genome sequence of *Malus kansuensis* (Rosaceae)

**DOI:** 10.1080/23802359.2020.1847622

**Published:** 2021-01-14

**Authors:** Lingli Li, Yang Ye, Mengran Zhao, Haodong Xin

**Affiliations:** a College of Forestry, Northwest A&F University, Yangling, PR China;; b Shaanxi Province Key Laboratory of Economic Plant Resources Development and Utilization, Yangling, PR China

**Keywords:** *Malus kansuensis*, chloroplast genome, phylogenetic analysis

## Abstract

The *Malus kansuensis* belongs to the *Malus* genus of Rosaceae family and it is an important apple rootstock resource native to China. Here, its complete chloroplast genome was sequenced and assembled by high-throughput Illumina sequencing data. The DNA was circular in shape with 160,133 bp length, containing IRA and IRB inverted repeat regions (26,354 bp), large single-copy region (LSC) (88,141 bp), and small single-copy region (SSC)(19,284 bp). In the chloroplast genome, 129 functional genes were predicted, including 85 protein-coding genes, 36 tRNA genes, and 8 rRNA genes. The phylogenetic tree basically accords with the traditional taxonomy of the order *Malus* genus of Rosaceae family.

The *Malus kansuensis* (Batalin) C. K. Schneid is a particular wild fruit tree native to China, which belongs to the *Malus* genus of Rosaceae family (Wang et al. 2013). This species has a well-developed main root system, highly resistant to stress, dwarfing habit, and early fruiting, so it is an important apple rootstock resource. In addition, this species fruits have high nutritional flavonoid content (Zhang et al. 2010). The complete chloroplast (cp) genome can help us reveal the evolutionary relationship of valuable plant species. In this study, the *M. kansuensis* cp genome was sequenced and assembled by Illumina sequencing data and we also analyzed its phylogenetic relationship in the *Malus* genus.

A single individual *M. kansuensis* (8-year-old) leaves were collected from the economic plants research base of the Northwest A&F University (Yangling Country, Shannxi Province, China; 34.262678 N, 108.069433 E). The specimens (WUK 0031609) had been preserved in the plant herbarium of the Northwest A&F University. The genomic DNA of the leaves using the DNeasy Plant Mini Kit (Qiagen, CA, USA). The whole-genome sequencing was sequenced by the Illumina HiSeq X Platform (Illumina Inc., San Diego, CA) with 150 bp pair-end reads. About 11.5 Gbp high-quality-sequencing reads were obtained. Using NOVOPlasty software (Dierckxsens et al. 2017), the cp genome was assembled. The resultant assembled cp genome was annotated based on the GeSeq (Tillich et al. 2017) and by comparing with cp genome of *Malus sieversii* MK434920. A graphical map of the annotated circular cp genome was generated using OGDRAW program (Lohse et al. 2013). The annotated complete cp genome of *M. kansuensis* has been submitted to GenBank (the accession number: MW018863).

The complete cp genome of the *M. kansuensis* was a double- stranded and circular DNA of 1,60,133 bp in length. The structure is similar to other *Malus* genus species which including IRA and IRB inverted repeat regions (26,354 bp), large single-copy region (LSC) (88,141 bp), and small single-copy region (SSC) (19,284 bp). In the cp genome, 129 functional genes were predicted, including 85 protein-coding genes, 36 tRNA genes, and 8 rRNA genes. Most of the functional genes appear in a single copy and 18 gene types appear in two copies or more, including the protein-coding gene (*rps7*, *rps12*, *rpl2*, *rpl23*, *ndhB, ycf1,* and *ycf2*), the tRNA genes (*trnA-UGC*, *trnI-GAU*, *trnL-CAA*, *trnM-CAU*, *trnV-GAC*, *trnR-ACG*, and *trnN-GUU*) and the rRNA genes (*rrn23*, *rrn16*, *rrn5*, and *rrn4.5*). The GC content of this species cp genome was 36.6%.

In order to investigate this cp genome phylogenetic placement in the *Malus* genus, the phylogenetic analysis was performed based on the protein-coding genes for 13 *Malus* genus species and the *Chaenomeles sinensis* as an outgroup (Figure 1). The phylogenetic tree was constructed using the maximum-likelihood method with 1000 bootstraps under the GTRGAMMAI substitution model. It was carried out using MEGA version 6 (Tamura et al. 2013). The phylogenetic tree basically accords with the traditional taxonomy of the order *Malus* genus of Rosaceae family. The cp genome of *M. kansuensis* can be further used for population genomic analysis for this important wild fruit tree species.

**Figure 1.**
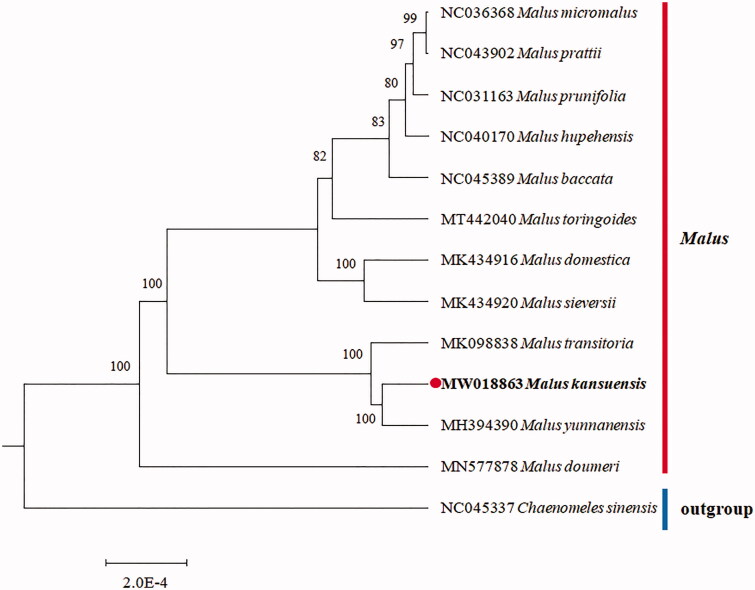
The phylogenetic tree is inferred from 13 chloroplast genomes. The *Malus kansuensis* complete cp genomes obtained in this study are shown in bold. Bootstrap values are shown at the branches.

## Data Availability

The data that support the findings of this study are openly available in GenBank with the accession number of MW018863 (https://www.ncbi.nlm.nih.gov/nuccore/ MW018863).
